# 肺癌相关成纤维细胞的研究进展

**DOI:** 10.3779/j.issn.1009-3419.2012.06.11

**Published:** 2012-06-20

**Authors:** 诚 沈, 国卫 车

**Affiliations:** 610041 成都，四川大学华西医院胸外科 Department of Thoracic Surgery, West China Hospital, Sichuan University, Chengdu 610041, China

**Keywords:** 肺肿瘤, 肿瘤微环境, 癌相关成纤维细胞, Lung neoplasms, Microenvironment, Cancer-associated fibroblasts

## Abstract

癌相关成纤维细胞（cancer-associated fibroblasts, CAFs）是肿瘤组织中被癌细胞激活的成纤维细胞，具有成肌纤维细胞的特性。CAFs表型恶性转换与肿瘤演进密切相关，同时CAFs是肿瘤微环境的主要组织成分。本文主要从以下方面结合肺癌进行综述：①CAFs的来源及其形态学特征，CAFs异质性及其表型时、空转换的临床意义；②CAFs的研究现状及临床应用中的问题；③CAFs与肺癌侵袭与转移的关系；④CAFs对肺癌预后及其治疗的影响。

## Abstract (en)

Cancer-associated fibroblasts (CAFs) is fibroblast cells activated by cancer cells in tumor with myofibroblasts cells (MFs) properties. CAFs is the main components of the tumor microenvironment. Malignant conversion of CAFs phenotype is closely related to the tumor evolution. In this review, we summarized that: (1) The source of CAFs and morphology, and the relation between heterogenicity and the malignant phenotype conversion of CAFs with time-space change; (2) The current research status and question of clinical application on CAFs; (3) The relation of CAFs and invasion and metastasis of lung cancer; (4) CAFs in the prognosis and treatment of lung cancer.

肿瘤演进的各个阶段均与其周围的环境进行交互对话（cross-talk），从而形成肿瘤-宿主界面微环境（microenvironment of the tumor-host interface）。但目前的研究与治疗主要集中在“种子-癌细胞”上，而对于“土壤-肿瘤微环境”中的分子机理了解甚少。然而，针对“种子-癌细胞”的“毒药”治疗效果差，迫使人们不得不重视对“土壤”的研究，以期为肿瘤的治疗开辟新的途径。现有研究^[1]^表明成纤维细胞表型转换也发生在组织的创伤和炎症修复过程中，且这种持续的刺激能促进上皮细胞的增生并最终形成癌细胞，癌细胞又进一步促进成纤维细胞表型变化。一般认为成纤维细胞表型转换是癌细胞分泌各种因子作用的结果，目的是创造一种适应或利于肿瘤生长、演进的微环境，即肿瘤微环境（tumor microenvironment, TME）^[1]^。研究癌相关成纤维细胞（cancer-associated fibroblasts, CAFs）表型和功能上转换的分子机制，有助于更好地了解肿瘤的生长环境，并有望成为肿瘤治疗的新靶点。

## 1 CAFs来源及其异质性

### 1.1 CAFs的来源及其形态学特征

癌相关成纤维细胞具有成肌纤维细胞（myofibroblasts, MF）的特性，其特征性的表型标记有α-平滑肌肌动蛋白（α-smooth muscle actin, α-SMA）、成纤维细胞活化蛋白（fibroblast-activating protein, FAP）、波形蛋白（Vimentin）。CAFs的主要来源有：①由宿主残存间质中的成纤维细胞在癌细胞分泌各种细胞因子，如转化生长因子-β（transforming growth factor-β, TGF-β）和血小板衍生因子（platelet-derived growth factor, PDGF）作用下诱导分化形成^[2]^；②上皮性肿瘤细胞通过上皮-间质转化（epithelial-mesenchymal transition, EMT）转化而来^[3]^；③CAFs与血管平滑肌细胞和血管外膜细胞有极大的相似性，也有可能是血管平滑肌细胞和血管外膜细胞分化而成^[4]^；④由骨髓基质干细胞直接转化^[5]^；⑤衰老的人成纤维细胞表型转换，如表皮生长因子（epidermal growth factor, EGF）和基质金属蛋白酶（matrix metalloproteinases, MMPs）的表达，其功能特性类似于CAFs，所以衰老的人成纤维细胞也可能是CAFs的前体细胞^[2]^。进一步通过倒置相差显微镜、透射电镜及免疫组化染色观察发现，CAFs的细胞呈纺锤形或长梭形，胞质突少，细胞大小不一致，生长密集，排列方式较杂乱，无方向性，出现重叠生长，接触和密度抑制丧失；胞核着色均匀，核仁明显。胞质内见大量粗面内质网、线粒体以及成束平行排列于胞膜下的肌丝，另见散在的电子致密斑，类似平滑肌的致密斑^[4, 6]^。而一般正常成纤维细胞（normal fibroblast, NF）呈多胞质突的扁平星状，细胞大小基本一致，排列有一定方向性；胞核形态不规则，胞质内富含粗面内质网，高尔基复合体发达，未见肌丝和致密斑；无重叠生长，具有接触抑制和密度抑制特征。CAFs与正常成纤维细胞的表型明显不同，α-SMA和Ⅰ型胶原表达明显增加，细胞增殖能力明显增强，与永生化的上皮细胞共同注入裸鼠，其成瘤能力高于单独接种上皮比较细胞500倍^[7]^。

### 1.2 CAFs的异质性及其表型时、空转换的含义

CAFs的异质性主要表现在其细胞表型不同，而其CAFs表型转换具有时、空特性：一是时相性，即CAFs表型转换与肿瘤发展阶段密切相关；二是空间特性，一方面是指癌组织不同部位的成纤维细胞具有不同的表型，另一方面是同一成纤维细胞在组织内不同部位呈现不同表型。研究发现由正常成纤维细胞转化为CAFs时，其表型转换与疾病演进过程密切相关。目前大家的共识是癌细胞与CAFs相互促进二者表型转换，共同参与肿瘤演进过程，且CAFs具有异质性^[1, 2]^，但对成纤维细胞异质性（表型变化的时、空特性）的原因尚不清楚。当前研究的争议有：①不同肿瘤中CAFs表型是否不同？②同一肿瘤CAFs与肿瘤细胞距离不同其表型也不同（空间特异性），其原因是癌细胞分泌相同细胞因子作用于不同亚型的成纤维细胞的结果，还是相同的成纤维细胞对癌细胞分泌的细胞因子选择性作用？③CAFs的表型转换是不是肿瘤细胞随时间“选择”的结果（时间特异性）。这些问题成为目前研究的热点和难点，越来越多的研究提示CAFs表型转换与肿瘤演进密切相关。

## 2 CAFs研究现状及临床应用中的问题

在Pubmed数据库中分别以“Cancer-associated Fibroblasts”和“lung cancer and Cancer-associated Fibroblasts”作为关键词并限定在Title（标题）中，截止到2012年2月29日，分别得到269篇和21篇研究文献（图 1A）。对这些文章的发表时间（图 1B）及类型分析发现：①文章数量在2011年增加，占总数的40%（118/294），而且近5年（2007年1月-2012年1月）的数量占到90%（264/294），2007年以前发表的文章数量只占10%左右（30/294）；②基础研究比例高达80%（224/294）（图 1C），临床研究只有9.8%（29/294）；③从文章发表的杂志看（图 1D）4种杂志发表数量占到所有杂志的38.77%（114/294），均是影响因子较高的杂志；④从CAFs与肿瘤类型看，目前的研究仍以乳腺癌、胰腺癌、前列腺癌和结直肠癌为主，肺癌只有21篇（8.4%）。结果提示：①CAFs的研究只是在最近1年-2年成为研究的热点，且多集中在基础研究；②研究集中于腺上皮来源的腺癌，肺癌的研究相对较少。上述文章研究结果的共识有：①CAFs与肿瘤细胞相互作用可加速肿瘤的演进过程；②CAFs表型的恶性转换与肿瘤患者预后差呈正相关；③CAFs通过多种信号通路与肿瘤细胞进行交互对话，但机制不清；④不同器官的肿瘤组织中的癌成纤维细胞表型不同，α-SMA的表达水平在不同肿瘤的CAFs中表达水平不同，如前列腺癌相关成纤维细胞的表达降低^[8]^。这些共识存在的问题有：①多数结论来自肿瘤细胞系的研究，结果难以应用于临床；②临床研究也局限于肿瘤标本，只有CAFs分子标记物表达水平与临床病理生理学特征及预后的关系，难以探讨其相互作用机理。

**1:**
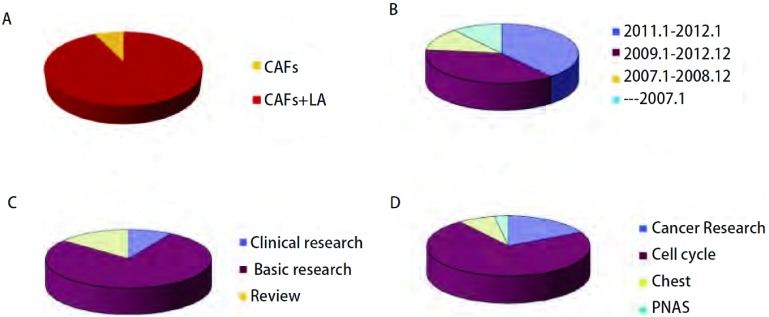
已发表的CAFs相关文章分析。A : CAFs与发表时间的关系；B：CAFs发表文章类型；C：CAFs发表杂志；D：CAFs与肿瘤种类。 The analysis of related published articles on CAFs. A: The published article number on CAFs and time; B: The published article types on CAFs; C: The published article number on CAFs and journals; D: The published article number on CAFs and tumor types. CAFs: cancer-associated fibroblasts.

## 3 CAFs与肺癌的侵袭与转移

CAFs在肿瘤血管内皮细胞的募集、活化、增殖和血管芽的形成中扮演着关键角色。乳腺癌、胰腺癌和前列腺癌等组织的CAFs表达的Fox蛋白（Forkhead box F1, FoxF1）、小窝蛋白-1（Caveolae-1, Cav-1）与趋化因子及其受体均参与内皮细胞活化和肿瘤血管新生过程。Fox蛋白是最新发现的一个转录因子家族，Foxf1在细胞增殖、分化和器官发育、塑型和功能形成中具有关键作用^[9, 10]^。Foxf1在肺的间质尤其是CAFs中大量表达^[11]^。Saito等^[12]^将肺癌细胞株A549细胞与Foxf1转导的NIH3T3细胞共培养，结果显示CAFs增强了A549的增殖能力，且Foxf1转导的成纤维细胞较普通成纤维细胞明显提高了A549肺癌细胞株的侵袭能力。*Cav-1*基因被认为是一个抑癌基因，在肺癌等多种恶性肿瘤中表达缺失，而癌细胞诱导CAFs中*Cav-1*基因表达缺失，进一步加速肿瘤生长与转移^[13]^。

Wald等^[14]^研究发现CAFs表达CXCL12能够明显促进前列腺癌细胞L3、L4和肺癌A549细胞株的生长能力及克隆形成率，且A549能力最强；但不同种类的肿瘤CAFs表达的CXCL12影响能力不同。肺癌细胞表达趋化因子受体CXCR和CAFs表达的CXCL12与肺癌器官特异性转移密切相关。Muller等^[15]^也证实肺癌的常见转移器官如骨、肝脏、肾上腺和脑中有高表达的CXCL12；CXCR4作为趋化因子CXCL12的受体，细胞膜高表达CXCR4的肿瘤比低表达的肿瘤更容易转移^[16]^。CXCL12参与肿瘤转移的机制或与表达CXCL12的CAFs细胞参与了血管的生成有关^[17]^。

Twist1（胚胎以发育过程中高度保守的基因）的再次激活可通过EMT途径促进肿瘤的发生与演进，且其在胃癌细胞和胃癌CAFs表达水平呈线性相关，提示CAFs的形成可能受肿瘤影响^[18]^。Twist表达上调可能引起肺泡上皮向肿瘤转化，Twist表达水平与肺癌的分化程度密切相关，研究^[18]^发现CAFs细胞中Twist1表达水平明显高于肿瘤细胞，提示CAFs与肿瘤细胞之间可能有协同效应，表达Twist1的CAFs可以促进表达Twist1的肿瘤细胞转移。Pallier等^[19]^研究发现肺腺癌中*EGFR*突变患者肿瘤细胞中*Twist1*基因表达升高，提示*Twist1*基因在肺癌细胞中的表达与*EGFR*的突变相关，并且与肺癌的恶性表型相关。

肾小球足突细胞粘蛋白（podoplanin）表达于毛细淋巴管内皮而不是血管内皮，是目前较为公认的肿瘤淋巴内皮标志物之一，已发现在多种肿瘤中表达并与肿瘤的淋巴转移相关。Hoshino等^[20]^研究112例人肺腺癌CAFs中Podoplanin表达，32例标本CAFs中的Podoplanin表达阳性，且均有血管侵袭和淋巴结转移。相关研究^[21, 22]^表明Cav-1的缺失也与淋巴结转移有关，而Cav-1的高水平表达可以减小肿瘤的转移。

## 4 CAFs对肺癌的预后及治疗的影响

肺癌的预后指标一直是困绕临床的难题，近来研究发现CAFs表型的恶性转换与患者的预后密切相关。肿瘤演进过程与CAFs和癌细胞细胞因子或基因表达水平呈正相关，且与肺癌患者预后呈负相关，如Podoplanin^[20]^和碳酸酐酶Ⅸ，肺腺癌间质纤维细胞中表达Podoplanin的患者预后较差并且容易早期复发^[23]^。Saito等^[12]^在研究中注意到，尽管表达Foxf1阳性的CAFs在所有人群中不能很明显地表现出与预后的关系，但局限于女性人群和大细胞肺癌的患者生存率很差。细胞因子在癌细胞与相关成纤维细胞中的表达水平明显不同（如趋化因子CXCL12/CXCR4表达阳性的癌细胞被表达阴性的CAFs包围，反之亦然），但CXCL12/CXCR4在肺癌组织及CAFs的表达均促进了非小细胞肺癌的演进，与患者预后呈负相关，认为肺癌的细胞膜表达的CXCR4可以作为非小细胞肺癌的预后指标。*Cav-1*基因表达降低或缺失与患者预后呈负相关^[24]^。在原发性非小细胞肺癌的CAFs中大量表达Twist1基因的患者预后差，该结果与Sung等^[18]^研究的结果一致。

由于CAFs可促进肿瘤的形成、生长、侵袭和转移，故将CAFs及其所产生的因子作为抗肿瘤治疗药物设计的靶点已成为可能。CAFs适合作为抗肿瘤治疗新靶点的原因有：①CAFs是非转化细胞，其基因组较肿瘤细胞稳定，不会出现抗原丢失和治疗耐受；②间质成分通常占肿瘤组织质量分数的50%-90%，是丰富的靶标结构；③CAFs通常靠近肿瘤间质中的血管内皮细胞并围绕癌巢；④间质细胞在各种肿瘤中的差异较小，针对肿瘤间质的抗肿瘤治疗可用于多种实体肿瘤。肺CAFs的特殊作用为治疗肿瘤提供了新的思路：①减弱或阻止传入CAFs的促活化信号，减少其分泌的各种因子，从而阻止肿瘤微环境中的血管形成和肿瘤细胞的发生、发展和转移。Tejada等^[25]^发现由肺癌细胞分泌的血小板源性生长因子（platelet-derived growth factor, PDGF）可以诱导肺癌间质中的CAFs生长和增殖。而相关文献^[26]^报道伊马替尼是血小板源性生长因子受体（platelet-derived growth factor receptor, PDGFR）的阻断剂，Kinoshita等^[27]^研究发现伊马替尼可以通过抑制CAFs中的PDGFR的络氨酸激酶活性从而抑制CAFs自身的生长，间接抑制CAFs对肿瘤的作用。Haubeiss等^[28]^在其研究中发现达沙替尼可以更加高效地抑制CAFs上的PDGFR的激酶活性来抑制肿瘤的生长，其机制可能是达沙替尼联合作用于PDGFR的不同激酶如Src激酶、TEC激酶、MAP激酶等^[29]^；②减弱或阻断由CAFs产生的各种因子或者蛋白的作用，Cheng等^[30]^发现应用成纤维细胞活化蛋白多克隆抗体处理人结肠癌细胞HT-29异种移植瘤后肿瘤生长减慢。目前各种生长因子、细胞因子抑制剂正投入临床各期试验中；③以CAFs为靶标促进肿瘤CAFs的死亡或凋亡。诱导CAFs程序性死亡也是抗肿瘤间质治疗的策略之一。目前，已设计出的精氨酸-甘氨酸-天冬氨酸多肽触发依赖半胱氨酸天冬酰胺特异蛋白酶的程序性死亡途径，降低了CAFs的生存率。另外，Pietras等^[31, 32]^发现实体瘤间质液压（interstitial fluid pressure, IFP）升高可妨碍抗肿瘤药物的有效运输，CAFs可通过PDGF调节IFP。体内动物研究证实PDGF受体拮抗剂可有效降低IFP，提高化疗药物的治疗效果。

## 5 展望

CAFs是非转化细胞，与肿瘤细胞相比其基因组较为稳定，因此破坏CAFs的生长或干扰其功能后，可通过阻碍新生血管形成而抑制肿瘤细胞的生长和侵袭。CAFs的异常增殖能力对上皮细胞的间质化等生物学特性产生影响，从而营造有利于肿瘤发生发展的肿瘤微环境。因此，CAFs恶性表型转换所需要的表达水平变化的Foxf1转录因子、*TWIST-1*基因、CXCR/CXCR4轴以及Podoplanin等都将成为肿瘤基因治疗或免疫治疗的新靶点，这种针对肿瘤微环境的治疗也将会成为肺癌防治的新途径。
